# Marine Microbial Diversity as a Source of Bioactive Natural Products

**DOI:** 10.3390/md18040215

**Published:** 2020-04-16

**Authors:** Didier Stien

**Affiliations:** Laboratoire de Biodiversité et Biotechnologie Microbiennes, Sorbonne Université, CNRS, LBBM, Observatoire Océanologique, 66650 Banyuls-sur-Mer, France; didier.stien@cnrs.fr

Some 3.5 billion years ago, microorganisms were the first to colonize Earth. They have gradually evolved, within intricate systems of microbial and macroscopic species, to occupy virtually all the available niches on the planet. Genetic drift and natural selection have molded the phenotypic expression of a trillion different microbial species [1], shaping their metabolisms and offering ever-more advantageous abilities to expand, with the production of new metabolites being one key to the fitness and evolutionary success of new species [2].

The rate of discovery of new natural products of microbial origin has increased significantly since the 1970s with the advent of modern methods of purification and structural determination, and later, with holistic approaches to chemical analysis and genetic information-based exploration of natural chemodiversity [3,4,5,6]. With these tools in hand and the exploration of innovative and ecologically relevant sources of microorganisms, it is possible to rapidly expand the exploration of chemodiversity on a global scale, and eventually isolate innovative compounds. It should be noted that the enthusiasm to discover new scaffolds is not necessarily relevant in the context of the search for active or useful natural products. Structural modifications that do not affect the core scaffold of a metabolite may provide a significant benefit that can be developed for a therapeutic use. A notable example is in the immense diversity of terpenes built on a limited number of scaffolds. Terpenes have different functional roles—and therefore different biological activities—and have largely contributed to the diversification of higher plants [2,7].

Today, marine microbes can still be regarded as a relatively underappreciated potential source of active compounds yet to be discovered. Some microorganisms can live freely in the water column, while many others live in association. For example, sponges host microorganisms that contribute to their holometabolome, while many coral species host microalgae and symbiotic prokaryotes. Furthermore, many microorganisms contribute to the construction of interspecific biofilms covering biotic and abiotic surfaces. While recent advances in natural-product science now provide access to an unknown dimension of chemodiversity, metabolic engineering will eventually allow the production of active marine compounds in amounts necessary for pharmaceutical development.

The Special Issue “Marine Microbial Diversity as a Source of Bioactive Natural Products” was aimed at collecting papers with up-to-date information regarding the characterization of marine microbes’ metabolic diversity and the evaluation of the therapeutic potential of marine microbes’ metabolites. The interest of the Special Issue was also to show that the exploration of underrated reservoirs of marine microorganisms can lead to the discovery of valuable secondary metabolites. In total, 10 articles were accepted and included in the Special Issue.

Most of the articles in this special issue deal with marine fungi, biological and chemical diversity, and their active metabolites. This may be a sign that marine fungi have been under studied to date, and are perceived by many researchers as an important source of discovery in this field [8]. Focusing on marine *Arthrinium* spp. isolates, Heo et al. conducted a phylogenetic analysis based on internal transcribed spacers, nuclear large subunit rDNA, β-tubulin, and translation elongation factor region sequences [9]. The 28 analyzed strains were obtained by cultivation of seaweed tissues (including several *Sargassum fulvellum* individuals) and *Arctoscopus japonicus* egg masses. It was found that the 28 isolates were in fact 15 species, 11 of which being new to Science. Most of the fungal extracts exhibited radical-scavenging activity, and some showed antifungal activity, tyrosinase inhibition, and quorum sensing inhibition. Interestingly, three species were found in both *S. fulvellum* and *A. japonicus* egg masses, perhaps because these fish usually lay eggs on *S. fulvellum*. The known compound gentisyl alcohol was found to be responsible for the radical-scavenging activity of two of the *Arthrinium* extracts. A fungal diversity analysis was also conducted on decommissioned salterns and neighboring mudflats in the Yellow Sea of South Korea [10]. Comparative fungal community analysis showed that the salterns that had been abandoned for more than 35 years had recovered to mudflats. The Yongyudo saltern was abandoned less than one year before the analysis and had not recovered. Its fungal community was more diverse and was dominated by Entorrhizomycota, while Chytridiomycota and Mortierellomycota dominated elsewhere. It has been hypothesized that Entorrhizomycota may include plant pathogenic fungi and that the dominance of this phylum may originate from the occurrence of their host plants at the initial stage of ecological succession after the saltern was abandoned. It should be mentioned that a large number of fungi could not be identified, indicating a lack of DNA-based phylogenetic information on marine fungi. Eventually, 53 fungal strains were isolated from the different sampling locations. The cultivable fungi were not necessarily the main taxa from the community analyses. A total of 18 isolates were possibly new species, and the authors provide the antioxidant, antifungal, tyrosinase inhibition, and quorum quenching activity of all isolates.

Fungal spores are easily dispersed and are detectable in community analyses. As a result, not all fungi detected in marine environments by rDNA-based analysis are genuine marine species. The challenging question of the isolation of marine fungi sensu stricto is key to the future exploration of the chemical diversity of marine fungi, and possibly the exploitation of their secondary metabolites. A best-practice guide for the isolation of marine fungi from different matrixes and their conservation is presented by Overy et al. [11]. Generalist, osmotolerant/halotolerant genera such as *Aspergillus* and *Penicillium* are highly cited in the literature but taxonomic groups composed predominantly of marine fungi sensu stricto such as Halosphaeriaceae, Torpedosporales, and Lulworthiales have been little studied for now. It should also be noted that mycologists have been studying marine fungi for decades, and that currently cryopreserved marine fungi may represent an easily accessible source for the exploration of their chemical diversity and the isolation of active secondary metabolites.

One of the sources frequently explored in the search for marine fungi are sedimentary deposits. Examination of the full genome of the marine-derived fungus *Penicillium brasilianum* HBU-136, isolated from the Bohai sea in China, highlighted that the strain harbors multiple non-ribosomal peptide synthetase (NRPS) biosynthetic gene clusters (BGC), including one showing high similarity to the BGC of fumitremorgins A-C in the fungus *Aspergillus fumigatus* [12]. The strain was already known to produce a spirocyclic diketopiperazine alkaloid and cyclotryprostatin B when cultivated on rice medium. More fermentation conditions were experimented and it was eventually discovered that three new and unusual indole-diketopiperazines **1**–**3** were produced by fermentation in a rice medium supplemented with 1% MgCl_2_ (Figure 1). The proposed BGC is likely responsible for the synthesis of these metabolites. Compound **1** was cytotoxic on HL-60 cells (6.0 µM), and compounds **2** and **3** were active on MCF-7 cell line (7.6 and 10.8 µM, respectively). The marine-derived fungus *Aspergillus fumigatus* CUGBMF170049 was also isolated from the Bohai sea sediments and was shown to produce new pseurotin analogs **4** and **5** (Figure 1) [13]. These metabolites were identified by thorough analysis of spectroscopic data, but unfortunately, were not active in the antimicrobial assays used in this work. Incidentally, the known compound helvolic acid isolated from the same fungus was very active on both *Staphylococcus aureus* and methicillin-resistant *S. aureus*, with a minimal inhibitory concentration (MIC) of 0.78 µg/mL. The fungus *Geosmithia pallida* FS140 was isolated from a sediment collected at 2403 m depth in the South China Sea [14]. Twelve diketopiperazines, including the three new thiodiketopiperazines geospallins A-C (**6**–**8**), were isolated from this fungus (Figure 1). The three new compounds exhibited substantial angiotensin-converting enzyme inhibitory activity, with IC_50_ values of 29–35 µM.

In the sea, many fungal species are associated with macroscopic organisms [9,11,15]. The isolation of marine fungi from macroorganisms’ tissues may in fact be a fairly straightforward way of isolating marine fungi sensu stricto. The fungus *Aspergillus flocculosus* 168ST-16.1 was isolated from the algae *Padina* sp., collected at a depth of 10 m in Da Nang, Vietnam [16]. Five new sesterterpene ophiobolins together with four known ones were isolated from a broth culture of this fungus and identified by spectroscopic methods. Interestingly, all these compounds were active against six cancer cell lines with 50% growth inhibition (GI_50_) values in the range of 0.14 to 2.01 µM. The new compound 14,15-dehydro-6-*epi*-ophiobolin K (**9**, Figure 2) was among the most active ones, with a GI_50_ of about 0.2 µM on all tested cell lines. The fungal strain *Botryosphaeria ramosa* L29 was isolated as an endophyte of the mangrove plant species. Four new isocoumarin derivatives, namely botryospyrones A, B (**10**), C, and D (Figure 2) were isolated from this fungus. Interestingly, a fifth compound (**11**) was produced only when the culture medium was supplemented with the host plant flavonoid (2*R*,3*R*)-3,5,7-trihydroxyflavanone 3-acetate, which had been found to slightly inhibit the endophyte growth. Owing to the ease of isomerization of animals by successive ring-opening and ring-closing steps, it is expected that such compound would eventually generate the more stable stereoisomer, i.e., the *cis* ring junction isomer. Further, the ^13^C NMR spectrum of compound **11** is similar enough to the one previously reported in the literature to conclude that the proposed structure was probably erroneous [17]. Compound **11** is in fact likely the (3a*R*,8a*S*)-1-acetyl-1,2,3,3a,8,8a-hexahydropyrrolo[2,3-*b*]indol-3a-ol rather than the (3a*S*,8a*S*) stereoisomer. Three coumarins and compound **11** were evaluated in vitro for antifungal activity toward three phytopathogenic fungi. It is interesting to point out that compound **11**, which is only produced by the fungus exposed to a metabolite of the plant, was the most active, with MIC values in the 28-57 µM range.

Finally, describing and understanding the metabolic diversity of marine species will allow a better understanding of the whole marine ecosystem, a better understanding of the mechanisms that regulate interspecific interactions in the sea, and possibly, a better understanding of the evolutionary processes involved. Moreover, the sea is the cauldron of a great diversity of useful and valuable compounds, whether known or not. As such, many marine microorganisms can become important sources of compounds useful to humankind. The carotenoid-producing yeast strain *Rhodotorula* sp. RY1801 was isolated from the exposed intertidal zone along the South Yellow Sea in Dongtai, China [18]. The culture conditions were optimized to increase the microbial biomass and the carotenoid yield of the strain. Eventually, it was possible to reach nearly 1 mg/L carotenoids in the culture, demonstrating that this fast growing *Rhodotorula* sp. strain may be used for the commercial production of carotenoids. The comparison of the phylogenetic and metabolomic profiles of 12 microalgal species from different lineages provided novel insights into the potential of chemotaxonomy in marine phytoplankton [19]. The most abundant and diversified metabolites identified over the 12 strains were polar lipids and pigments. Historically, analysis of pigment composition has been used to assist the classification of microalgae. Here, it was found that lipid classes and some specific lipids within classes may serve as phylogenetic markers. For example, within the tested Mamiellales, the *Ostreococcus* genus differs by the presence of two monogalactosyldiacylglycerols (MGDG 20:5/16:3 and 16:1/16:1), and the species *O. tauri* has six 1,2-diacylglyceryl-3-*O*-4’-(*N*,*N*,*N*-trimethyl)-homoserines (DGTSs) that are not found in *O. mediterraneus*. Overall, a good overlap of phylogenetic and chemotaxonomic signals was demonstrated, in particular when the analysis focused on the major metabolites of algae, and evolutionary divergence between species could be inferred in good congruence with the phylogenies. These results support the hypothesis of a metabolomics equivalent to the “molecular clock” based on the analysis of sequence data.

As seen from the above synopsis, the papers included in this Special Issue provide an interesting overview of chemical diversity of marine fungi and algae. New, bioactive, marine-derived fungal metabolites were isolated and characterized. The articles presented in this Special Issue underline the central role of the sea as a provider of valuable chemicals for human use.

In conclusion, the Guest Editor thanks all the authors who contributed to this Special Issue, all the reviewers for evaluating the submitted manuscripts, and the Editorial board of Marine Drugs, especially, Orazio Taglialatela-Scafati, Editor-in-Chief of the journal, and Estelle Fan, Assistant Editor, for their kind help in bringing this book into reality.

## Figures and Tables

**Figure 1:**
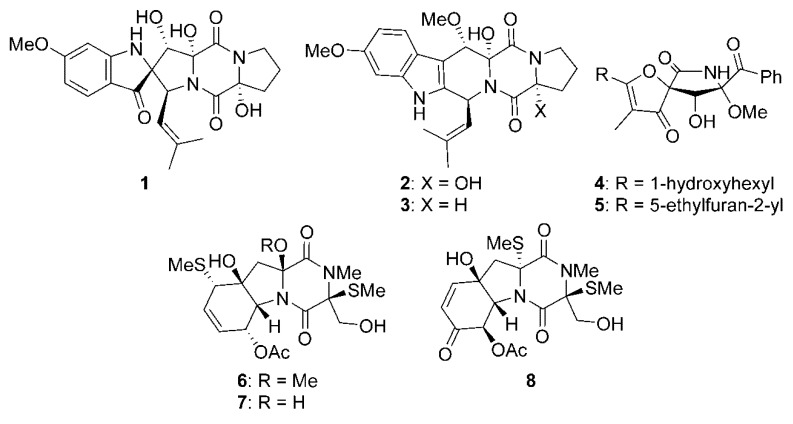
Compounds isolated from marine-sediment-derived fungi.

**Figure 2:**
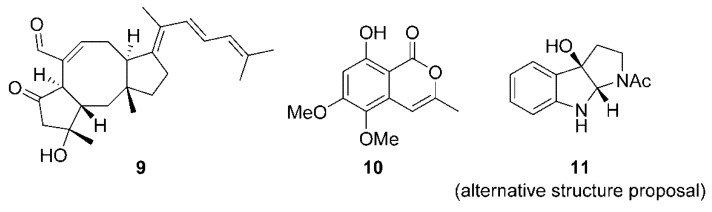
Examples of secondary metabolites isolated from fungi associated to algae and plants.
